# A Study of the Activity of Recombinant Mn-Superoxide Dismutase in the Presence of Gold and Silver Nanoparticles

**DOI:** 10.1007/s12010-018-2896-y

**Published:** 2018-10-03

**Authors:** Agnieszka Małgorzata Pudlarz, Katarzyna Ranoszek-Soliwoda, Ewa Czechowska, Emilia Tomaszewska, Grzegorz Celichowski, Jarosław Grobelny, Janusz Szemraj

**Affiliations:** 1 Department of Medical Biochemistry, Faculty of Health Sciences with the Division of Nursing and Midwifery, Medical University of Lodz, Mazowiecka 6/8, 92-215 Lodz, Poland; 2 Department of Materials Technology and Chemistry, Faculty of Chemistry, University of Lodz, Pomorska 163, 90-236 Lodz, Poland

**Keywords:** Superoxide dismutase, Expression, Gold nanoparticles, Silver nanoparticles, Immobilization

## Abstract

Superoxide dismutase (SOD) is one of the best characterized enzyme maintaining the redox state in the cell. A bacterial expression system was used to produce human recombinant manganese SOD with a His-tag on the C-end of the protein for better purification. In addition, gold and silver nanoparticles were chemically synthesized in a variety of sizes, and then mixed with the enzyme for immobilization. Analysis by dynamic light scattering and scanning transmission electron microscopy revealed no aggregates or agglomerates of the obtained colloids. After immobilization of the protein on AuNPs and AgNPs, the conjugates were analyzed by SDS-PAGE. It was determined that SOD was adsorbed only on the gold nanoparticles. Enzyme activity was analyzed in colloids of the gold and silver nanoparticles bearing SOD. The presence of a nanoparticle did not affect enzyme activity; however, the amount of protein and size of the gold nanoparticle did influence the enzymatic activity of the conjugate. Our findings confirm that active recombinant human superoxide dismutase can be produced using a bacterial expression system, and that the enzyme can be immobilized on metal nanoparticles. The interaction between enzymes and metal nanoparticles requires further investigation.

## Introduction

During the respiratory processes, reactive oxygen species (ROS) are created in the cells of eukaryotic and prokaryotic organisms. Their concentration is controlled enzymatically, e.g., by superoxide dismutase and catalase, as well as by non-enzymatic systems such as glutathione and ascorbate. ROS have many signaling and information functions; however, disturbances in the redox state of the cell may lead to excessive production of ROS, resulting in the peroxidation of lipids, oxidation of proteins and DNA damage and ultimately, cell death [1–5].

One of the key antioxidant enzymes is superoxide dismutase (SOD; EC 1.15.1.1), which structure and sequence are highly conserved. The eukaryotic organism possesses three isoforms according to their place of occurrence: cytoplasmatic (SOD1, Cu/ZnSOD), mitochondrial (SOD2, MnSOD), and extracellular (SOD3, EC-SOD). And at the active center of the enzyme, a manganese or copper and zinc ions can be found depending on the isoform [6, 7]. SOD catalyzes the conversion of O_2_^−^ into H_2_O_2_, which is then converted by catalase or peroxidase to harmless H_2_O and O_2_ [6–8]. In addition, mitochondrial SOD is also closely associated with the maintenance of energetic homeostasis in mitochondria and is believed to inform the cell of the need to activate alternative energy-producing pathways in the case of mitochondrial respiration breakdown [8]. It has been demonstrated that supplied SOD helps in wound healing [9, 10], protects against UV radiation [11, 12], and inhibits the proliferation of cancer cells [13]. It has been also shown that overexpression of SOD suppressed the malignancy of several types of cancers in vitro [14–16]. SOD has demonstrated a protective function against radiation and to have chemopreventive effects in in vitro and in vivo cancer models [17–20].

To determine the possible therapeutic effect of these proteins, an overexpression protein system and good-quality purification process are needed to produce them in large amounts. The most popular system is the *Escherichia coli* bacterial expression system, because of its relatively low cost of culture and large amount of protein production. In addition, *E. coli* are well characterized metabolically and physiologically [21]. This approach also offers the possibility of introducing recombinant modifications in the proteins, such as adding tags for more efficient purification. Such bacterial expression systems have been used to produce several types of recombinant human proteins which showed properties characteristic for original human proteins [22–24].

Although SOD offers great therapeutic potential, it has to be delivered into the organism through the digestive system or the circulatory system; such proteins are highly susceptible to digestion by proteolytic enzymes and are quickly removed from the circulation [25]; however, these disadvantages could be resolved with the use of a suitable drug delivery system [25, 26]. One of such delivery system acts by the immobilization of proteins on the surface of metal nanoparticles [27]. Two strong candidate materials for producing such nanoparticles are gold (Au) and silver (Ag). Range of favorable physical and biological properties of gold and silver nanoparticles are less than 100 nm in diameter and possess high surface to volume ratio, low toxicity, and high biocompatibility [27]. Because of their chemical properties, gold and silver nanoparticles are able to interact with many substrates and hence could be coated with a range of therapeutics [28]. Gold nanoparticles have been used as delivery system for chemotherapeutic agents [29], small interfering RNAs (siRNA) [30], and plasmid DNA [31] inside the cell. Also it has been demonstrated that proteins and peptides can be carried into cells following immobilization on the surface of gold nanoparticles [32]. Likewise, silver nanoparticles have a number of antimicrobial, antifungal, or even anticancer properties [33]; it is known that AgNP could generate undesirable reactive oxygen species, which could be reduced by some modifications of those nanoparticles [34, 35]. However, despite their biocompatibility, high surface to volume ratio and possibility to penetrate cells [33], like gold nanoparticles, silver nanoparticles have been rarely used as a drug delivery system [36].

The present article describes the production of human recombinant manganese SOD in an *E. coli* bacterial expression system. After obtaining the appropriate amount of purified protein, the enzyme was adsorbed onto the surface of gold (AuNP) and silver (AgNP) nanoparticles. Four size variants of AuNPs (13, 20, 31, and 42 nm) and four size variants of AgNPs (13, 27, 33, and 45 nm) were used for the experiment. SOD was mixed with the gold and silver nanoparticles at three different concentrations to obtain 66, 100, and 133% of coverage of the nanoparticle surface. The enzymatic activity of recombinant SOD in the presence of gold and silver nanoparticles was compared with the activity of recombinant SOD in aqueous solution. The immobilization state of SOD on gold and silver nanoparticles was determined by gel electrophoresis.

The recombinant human manganese SOD obtained in this process could be used in further studies. As a therapeutic agent, it could be used in healing wounds or in other diseases related with oxidative stress. The use of gold or silver nanoparticles could improve the delivery of SOD to cells and protect it from digestion. However, further studies on colloids of nanoparticles and adsorbed enzymatic proteins have to be performed.

## Materials and Methods

### Plasmid Construction

A plasmid with a human recombinant manganese SOD sequence (Gene Bank accession no. NM_000636) was constructed by amplifying SOD cDNA with the following primers: forward CTAGC*AAGCTT*CCATGTTGAGCCGGGCA and reverse: 5′GTTGC*ACGCGT*CTTTTTGCAAGC3′. The primers encompass the restriction enzymes sites HindIII (forward) and MluI (reverse), given in italics. The resulting amplified DNA fragments (690 bp) were digested with appropriate restriction enzymes (HindIII and MluI) and cloned into pEX-C-His (OriGene) under T7 promoter. The resulting plasmid contained the cDNA of human manganese SOD with a His-tag on the C-end of the protein. The plasmid was then transferred into *E. coli* XL-stain to identify the best clones by growth on plates with ampicillin selection medium. Plasmids were isolated from several of the resulting colonies, purified by PureYield™ Plasmid Miniprep System kit (Promega), and sequenced. The best constructs were transferred to *E. coli* BL-STAR stain for protein expression.

### Recombinant SOD Expression, Isolation, and Purification

BL-STAR strain *E. coli* with the pEX-SOD plasmid were grown overnight in LB medium with ampicillin at 37 °C. The inoculum was added to 100 ml LB medium with ampicillin, and the bacteria were grown at 37 °C till OD600 reached 0.4. IPTG was then added to make a final concentration of 1 mM. The bacteria were grown for 2 h at 42 °C, and then overnight at 22 °C. After growing, the cells were harvested by 20 min of centrifugation at 4000×*g* and stored at − 80 °C until protein isolation.

The bacterial pellet was suspended in B-PER™ Bacterial Protein Extraction Reagent (ThermoFisher Scientific) with 5 mg/ml lysozyme and incubated on ice for 20 min. After incubation, the bacteria were disrupted by sonication. Centrifugation was carried out for 20 min at 4000×*g*, the supernatant was stored, and the pellet resuspended in Inclusion Body Solubilization Reagent for 1 h on ice. The unresuspended molecules were centrifuged for 20 min at 4000×*g*.

Recombinant human manganese superoxide dismutase isolated from the soluble protein fraction and the inclusion bodies was purified by affinity chromatography using HisPur Ni-NTA Spin Columns (Thermo Science) under native and denaturating conditions, respectively, according to the manufacturer’s instructions. Mn-SOD purified from inclusion bodies was dialyzed against 25 mM Tris-HCl (pH 7,5), 150 mM NaCl buffer overnight at 4 °C then dialyzed against pure water under the same conditions. SOD purified in native conditions was also dialyzed against pure water at 4 °C. The extracts of the purified and dialyzed SOD were then combined and either taken for analysis or lyophilized until the beginning of the next step of the study.

### Superoxide Dismutase Activity Test

The activity of SOD in the presence of gold and silver nanoparticles and in aqueous solution was determined by the Superoxide Dismutase Activity Colorimetric Assay Kit (Abcam, ab65354) according to the manufacturer’s instructions. Activity was measured after purification; and after adsorption on nanoparticles, these readings were also followed up after 1 h, 6 h, 12 h, 24 h, 3 days, 6 days, 9 days, 12 days, 15 days, and 18 days. SOD activity in aqueous solution was also measured at these time points. During the experiment, SOD samples were stored at room temperature. Briefly, the assay was based on the activity of xanthine oxidase (XO); this produces a superoxide anion which reduces a tetrazolium salt (WST-1) to produce a water-soluble formazan dye. SOD reduces the level of superoxide anion converting it to oxygen and hydrogen peroxide, thus inhibiting WST-1 reduction. The activity of SOD was detected by colorimetry at 450 nm using an GloMax®-Multi Microplate Multimode Reader (Promega).

### SDS-PAGE and Western Blot

Protein samples were analyzed by 10% SDS-PAGE, followed by Coomassie Brilliant Blue staining for visualizing the purity of protein. First, the protein samples were mixed with sample buffer containing 62.5 mM Tris-HCl, pH 6.8, 25% glycerol, 2% SDS, 0.01% Bromophenol Blue, and 5% β-mercaptoethanol and denatured for 7 min at 95 °C. Electrophoresis was carried out for 1.5 h at 120 V (constant voltage).

The same parameters of electrophoresis were applied for Western blot analysis. After electrophoresis, the protein was transferred from the gel to a polyvinylidene fluoride (PVDF) membrane by an eBlot Protein Transfer Device (GeneScript). Then the membrane was incubated in 5% milk in TBS (Tris-buffered saline) for 1 h for blocking. Incubation with primary monoclonal mouse anti-histidine tag:HRP antibodies (Bio-Rad) was performed overnight at 4 °C. Finally, the membrane was washed with TBS-Tween 0.05% and developed with chemiluminescence using an ECL Western Blotting Substrate (Thermo Science).

Protein concentration was determined with BCA Protein Assay Kit (Thermo Science).

### Silver Staining

Protein immobilization was confirmed by SDS-PAGE, followed by silver staining for protein visualization according to the protocol already described in our previous work [37]. Bio-Rad Mini-Protean Tetra Cell was used for one-dimensional electrophoresis and the measurement was performed under constant conditions (38 min at 200 V, 10% acrylamide gel of 1 mm thickness). After electrophoresis, the gels were silver-stained according to a modified Shevchenko protocol [37, 38]. The intensity of each protein band was estimated digitally by scanning the gel with a densitometer (Molecular Imager Gel Doc XR+) and using imaging software (Image Lab 2.0, Bio-Rad Lab.).

### Nanoparticle Synthesis

The colloids were synthesized by two methods, the first being chemical reduction (colloids: AuNPs 13 nm, AgNPs 27 nm, AgNPs 33 nm, and AgNPs 45 nm) and the second being seeded-mediated growth (colloids: AuNPs 20 nm, AuNPs 31 nm, AuNPs 42 nm, and AgNPs 53 nm). The final concentration of metal in each colloid was 100 ppm.

## Gold Nanoparticles

### AuNPs 13 nm

An aqueous solution of chloroauric acid (95.4 g, 1.81·10–2% wt.) was heated under reflux to boiling point and sodium citrate solution was added (4.6 g, 1% wt.). The mixture was then boiled for 15 min and then cooled to room temperature.

### AuNPs 20 nm

A seed solution (20.6 g of AuNPs 13 nm colloid), deionized water (23.7 g), and sodium citrate solution (4%, 0.7 g) were heated to boiling under reflux. Next, a precursor of gold atoms (15.00 g, 0.0453% of chloroauric acid aqueous solution) was added to the reaction mixture with a constant flow rate of 12 ml·h^−1^. The mixture was then boiled for 15 min and cooled to room temperature.

### AuNPs 31 nm

A seed solution (6.1 g of AuNPs 13 nm colloid), deionized water (37.9 g), and sodium citrate solution (4%, 1.0 g) were heated to boiling under reflux. The precursor (15.00 g, 0.0620% of chloroauric acid aqueous solution) was then added to the reaction with a constant flow rate of 10 ml·h^−1^. The reaction mixture was heated for the next 15 min and cooled.

### AuNPs 42 nm

A precursor of gold atom (15.00 g, 0.0661% of chloroauric acid aqueous solution) was added to the reaction mixture (flow rate of 10 ml·h^−1^) containing seed solution (2.1 g of AuNPs 13 nm colloid), deionized water (34.5 g), and sodium citrate solution (4%, 0.9 g) heated to boiling under reflux. The reaction solution was heated for 15 min and then cooled to room temperature.

## Silver Nanoparticles

### AgNPs 13 nm

Seed synthesis was performed; thus, a mixture of sodium citrate (4.2 g, 4% wt.) and tannic acid (0.6 g, 5% wt.) was incorporated into 94.5 g of silver nitrate aqueous solution (1.66·10^−2^% wt.). A solution of sodium borohydride (0.7 g, 2% wt.) was then added to the reaction flask over the course of a few seconds and the solution was mixed for 15 min.

Nanoparticle synthesis—seed solution (2.7 g), deionized water (37.3 g), and sodium citrate solution (4%, 2.0 g) were heated to boiling under reflux. Next, the silver atom precursor (8.0 g, 0.122% of silver nitrate aqueous solution) was added to the reaction flask with a constant flow rate of 8 ml·h^−1^ and the mixture was boiled for the next 5 min.

### AgNPs 27 nm

95.2 g of silver nitrate aqueous solution (1.65·10–2% wt.) was heated under reflux to boiling point and a mixture of sodium citrate (4.2 g, 4% wt.) and tannic acid (0.6 g, 5% wt.) were incorporated to the reaction solution. The mixture was heated for an additional 15 min and cooled.

### AgNPs 33 nm

A mixture of sodium citrate (4.2 g, 1% wt.) and tannic acid (0.6 g, 5% wt.) was incorporated into silver nitrate solution (1.65·10^−2^% wt., 95.2 g) under reflux and heated for 15 min. The reaction solution was then cooled to room temperature.

### AgNPs 45 nm

A mixture of sodium citrate (4.2 g, 1% wt.) and tannic acid (1.3 g, 5% wt.) was added to an aqueous solution of silver nitrate (1.66·10^−2^% wt., 94.6 g) heated under reflux. The solution was boiled for an additional 15 min and cooled.

## Gold and Silver Nanoparticle Characterization

### Dynamic Light Scattering and Scanning Transmission Electron Microscopy

Gold and silver nanoparticles were characterized with Scanning Transmission Electron Microscopy (STEM) and Dynamic Light Scattering (DLS). STEM investigations were performed on an FEI NovaNanoSEM 450 (accelerating voltage 30 kV) by deposition of colloid on a carbon-coated copper grid. DLS measurements were performed on a Nano ZS zetasizer system (Malvern Instruments) using the following settings: laser wavelength of 633 nm (He–Ne); scattering angle 173°; measurement temperature 25 °C, medium viscosity 0.8872 mPa·s; medium refractive index 1.330, quartz microcuvettes. Colloids were filtered with 0.2 μm polyvinylidene fluoride (PVDF) membrane before measurements.

### Gold and Silver Nanoparticle Modification with Recombinant Human Superoxide Dismutase

The AuNPs and AgNPs were modified by the addition of recombinant human SOD by ligand exchange. This process was carried out by incubation of the NPs with protein. The percentage coverage of nanoparticles (66, 100, or 133%) was set by choosing an appropriate concentration of SOD (Tables 1 and 2). A mixture of the AuNP or AgNP colloid and an aqueous solution of protein were incubated at room temperature for at least 2 h until the protein was adsorbed on the nanoparticle surface. No filtration or postprocessing steps were carried out for any colloid after the modification process.

**Table 1:** The amount of superoxide dismutase used for the modification of AuNPs

NP size [nm]	Surface coverage [%]	Amount of SOD		Sample
		[SOD molecule/NP]	[g/ml]	
13	66	45	7.41·10^−6^	As1Au
	100	65	1.07·10–5	As2Au
	133	85	1.40·10–5	As3Au
20	66	105	4.75·10^−6^	Bs1Au
	100	150	6.78·10^−6^	Bs2Au
	133	195	8.81·10^−6^	Bs3Au
32	66	245	3.28·10^−6^	Cs1Au
	100	355	4.75·10^−6^	Cs2Au
	133	465	6.23·10^−6^	Cs3Au
43	66	440	2.49·10^−6^	Ds1Au
	100	630	3.56·10^−6^	Ds2Au
	133	820	4.63·10^−6^	Ds3Au

**Table 2:** The amount of superoxide dismutase used for the modification of AgNPs

NP size [nm]	Surface coverage [%]	Amount of SOD		Sample
		[SOD molecule/NP]	[g/ml]	
13	66	35	1.17·10^−5^	Es1Ag
	100	50	1.67·10^−5^	Es2Ag
	133	65	2.17·10^−5^	Es3Ag
27	66	150	5.07·10^−6^	Fs1Ag
	100	215	7.27·10^−6^	Fs2Ag
	133	280	9,46·10^−6^	Fs3Ag
33	66	225	3.07·10^−6^	Gs1Ag
	100	320	5.92·10^−6^	Gs2Ag
	133	415	5.69·10^−6^	Gs3Ag
45	66	420	3.07·10^−6^	Hs1Ag
	100	600	4.38·10^−6^	Hs2Ag
	133	780	5.69·10^−6^	Hs3Ag

## Results and Discussion

### Gold and Silver Nanoparticle Characterization

**Fig. 1:**
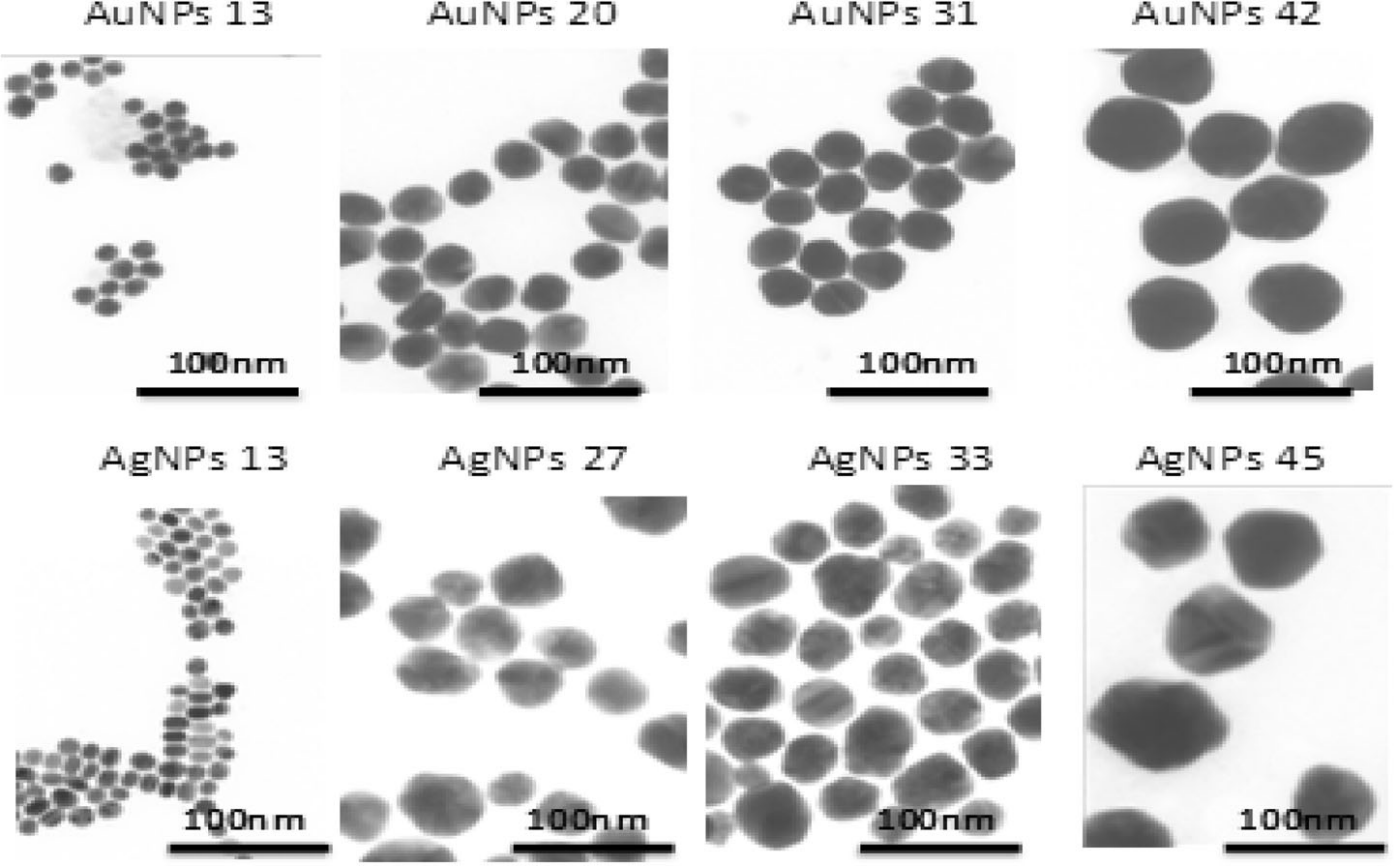
STEM images of gold and silver nanoparticles

Gold and silver nanoparticles were synthesized in aqueous solution. Gold nanoparticles with a diameter ranging from 13 to 42 nm were obtained chemically, with the size depending on the molar ratio of chloroauric acid and sodium citrate. Silver nanoparticles were in four sizes ranging from 13 to 45 nm. Both types of nanoparticles were characterized by DLS and STEM techniques (Table 3, Fig. 1). Nanoparticle size was determined by STEM images (Fig. 1). The size of the metallic core for each colloid was about 13, 20, 31, or 42 nm for the AuNPs and 13, 27, 33, or 45 nm for the AgNPs. The hydrodynamic size of the particles for each colloid was determined by DLS (Table 3). Neither STEM nor DLS analysis revealed any aggregates or agglomerates.

**Table 3:** The overall results of AuNP and AgNP characterization

Sample	STEM *d* [nm]	DLS *d*_H_ [nm]
AuNPs 13	13	17 ± 3
AuNPs 20	20	24 ± 4
AuNPs 31	31	37 ± 7
AuNPs 42	42	48 ± 9
AgNPs 13	13	18 ± 3
AgNPs 27	27	38 ± 6
AgNPs 33	33	49 ± 9
AgNPs 45	45	61 ± 11

*d* the size of metallic core of nanoparticles, *dH* hydrodynamic diameter of nanoparticles

### Production and Purification of Superoxide Dismutase

Human manganese SOD cDNA was cloned into a pEX plasmid containing a His-tag. This resulted in the creation of recombinant Mn-SOD protein with six histidine tags on the C-end. In the constructed plasmid, the open reading frame (ORF) was found to comprise 740 bp. *E. coli* were transformed with the recombinant pEX-SOD plasmid by thermal shock, and these were used for further screening and expression, as described in the “Materials and Methods” section.

**Fig. 2:**
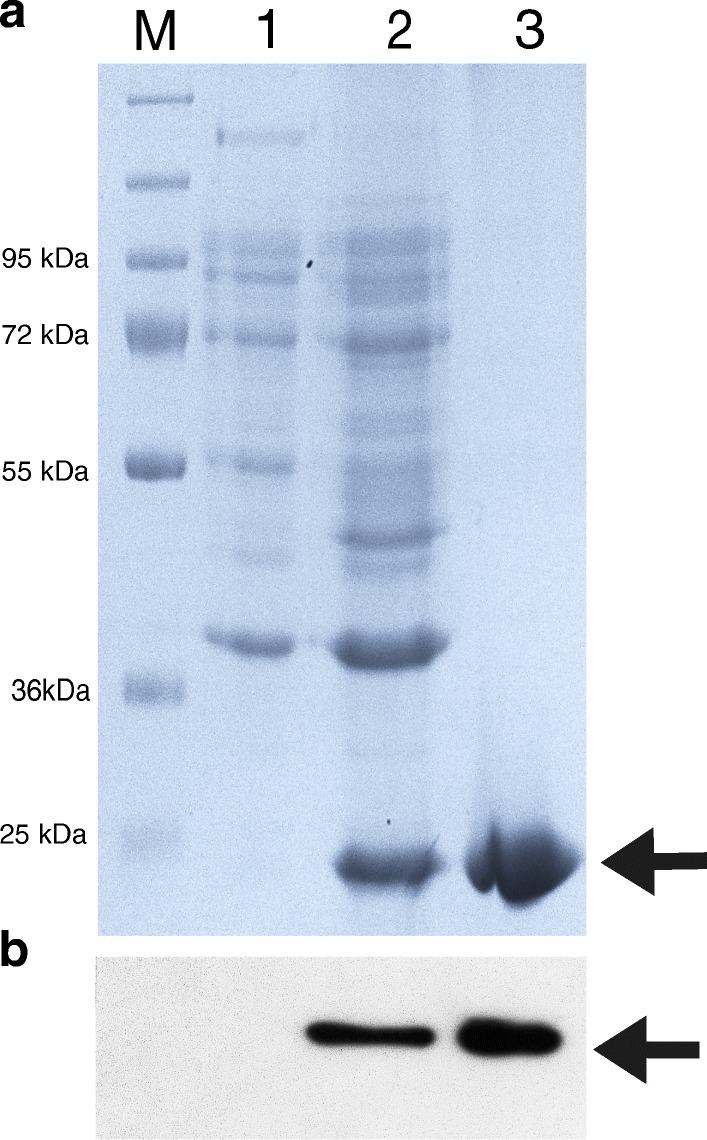
SDS-PAGE and Western blot analysis of expression of recombinant human SOD in the *E. coli* expression system. **a** SDS-PAGE of Mn-SOD expression: line 1: total protein from bacteria before induction with IPTG and temperature, line 2: total protein from night culture after induction with IPTG and temperature, line 3: SOD after purification, M: marker; **b** western blot analysis of SOD expression, line 1 protein from bacteria before induction, line 2: protein from bacteria after induction, line 3: purified SOD

A stein of *E. coli* BL-star (InvitrogenTM) containing the pEX-SOD functional plasmid were subjected to induction with IPTG at 42 °C for 2 h and then transferred to 22 °C and grown through the night. The bacteria were harvested by centrifugation, and the bacterial lysate was prepared for protein isolation and purification. The expression and purification of recombinant human SOD were analyzed by SDS-PAGE and Western blot (Fig. 2). SOD was detected as a monomer with a mass of 28 kDa. Purification was performed in native and denaturating conditions with 6 M guanidine on Ni-NTA spin columns (Thermo) to purify as much protein as possible. SOD containing the His-tag was removed from resin with 300 mM imidazole for native purification, and with 300 mM imidazole and 6 M guanidine for denaturating condition. The elution fractions were combined in two sets (native and denaturized) and dialyzed. The SOD in the denaturing buffer was first dialyzed against refolding buffer to renature and restore enzyme activity, and then both preparations were dialyzed against pure water.

From the culture, a 6.5 g bacterial pellet was obtained, from which was extracted 15.2 mg of protein (soluble and insoluble). From the whole protein extract, 3.4 mg of recombinant manganese SOD was purified. After dialysis, the activity of manganese SOD was determined to be approximately 10 U/mg. This low SOD activity could be caused by low dimer and tetramer formation, which is crucial for MnSOD activity [39]. This could be associated with the presence of denaturing agent even after dialysis.

### Modification of Gold and Silver Nanoparticles with Superoxide Dismutase

**Fig. 3:**
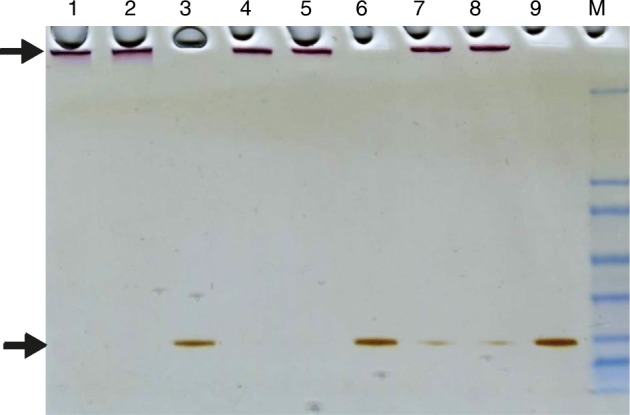
SDS-PAGE of immobilized SOD on gold nanoparticles of size 13 nm. Line 1,2: SOD on AuNP at 66% of surface coverage, line 3: amount of SOD corresponding to 66% of nanoparticle coverage, line 4,5: SOD on AuNP at 100% of surface coverage, line 6: amount of SOD corresponding to 100% of nanoparticle coverage, line 7,8: SOD on AuNP at 133% of surface coverage, line 9: amount of SOD corresponding to 133% of nanoparticle coverage, line M: marker

The ability of proteins and nanoparticles to create conjugates depends on the size and curvature of the nanoparticle, its morphology, crystal structure, and surface chemistry [40]. Recombinant human manganese SOD was immobilized on gold and silver nanoparticles by the ligand exchange method. The enzyme was mixed with gold and/or silver nanoparticles at three different concentrations variant to obtain 66, 100, and 133% of coverage of the nanoparticle surface. Gold nanoparticles were synthesized in four different sizes (13, 20, 32, and 43 nm) and were incubated until the protein was adsorbed on their surface. SDS-PAGE examination confirmed that SOD was immobilized on the gold nanoparticles (Fig. 3) and was similar for all nanoparticle sizes; an electrophoresis image of the SOD immobilized on 13-nm gold nanoparticle is given as the representative example (Fig. 3). The SOD immobilized on the nanoparticles can be seen in the upper part of the gel, while the unbound SOD can be seen at the bottom of the gel on the height of 28 kDa. It has been earlier demonstrated that gel electroforesis and protein detection by silver staining is an effective method of detecting grade of immobilization of protein on nanoparticles [37].

**Fig. 4:**
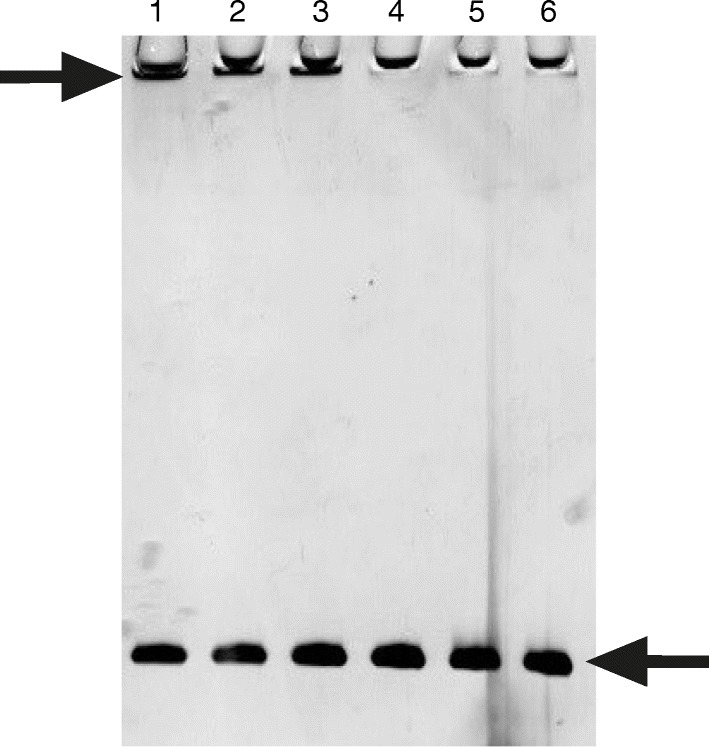
SDS-PAGE of SOD after adsorption on 13-nm silver nanoparticles with 66% coverage. Line 1,2,3: SOD and silver nanoparticles (pointed with arrow on top of the gel), line 4,5,6: SOD in corresponding concentration

Silver nanoparticles in all variants of dimensions (13, 27, 33, and 45 nm) were incubated with SOD for immobilization. It is difficult to state with any certainty that the SOD was immobilized on the silver nanoparticles. Figure 4 presents a gel with samples of 13-nm silver nanoparticle mixed with SOD corresponding to 66% of nanoparticle coverage. This is a representative example for all variants of size of silver nanoparticles and SOD concentrations. Previously, lysozyme and α-chymotrypsin were immobilized on gold nanospheres with a maximal final coverage of 30 and 20%, respectively [40]. Recent studies found that human serum albumin is significantly destabilized by silver nanoparticles but not gold nanoparticles [41]. Also studies on immobilization of amylase on gold nanoparticles show that only 20% of protein adsorbed on nanoparticles [42]. It has been shown also that immobilization of lysozyme on AgNPs is most efficient when the weight ratio of the enzyme to nanoparticles was 1:100; the immobilization efficiency decreased as the ratio increased [43].

Those results show that immobilization of the enzymes on the surface of metal nanoparticles is specific for each protein and nanoparticle. The interactions between proteins and nanoparticles have to be investigated more closely to understand and characterize those interactions.

### Superoxide Dismutase Activity Test in the Presence of Gold Nanoparticles

**Fig. 5:**
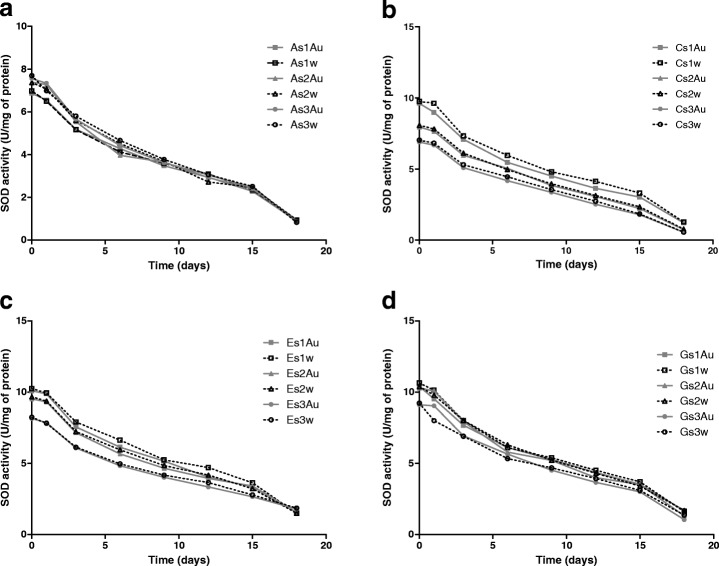
Activity of superoxide dismutase on gold nanoparticles (gray line) and in aqueous solution (black dotted line) for three variants of coverage (66% square, 100% triangle, 133% circle) during 18 days of experiment. **a** SOD on AuNPs of size 13 nm. **b** SOD on AuNPs of size 20 nm. **c** SOD on AuNPs of size 32 nm. **d** SOD on AuNPs of size 43 nm

Mn-SOD was added to colloids of gold nanoparticles at appropriate concentrations to obtain 66, 100, and 133% of nanoparticle coverage for all variants of nanoparticle size (13, 20, 32, and 43 nm). Small differences in SOD activity were observed between probes in aqueous solution which came from high dilutions of the protein. Activity tests found that SOD had activity after immobilization on gold nanoparticles (Fig. 5), and demonstrated similar activity in the presence of gold nanoparticles and in aqueous solution. Previous studies indicate that thermostable Mn-SOD immobilized on superparamagnetic nanoparticles at an optimal temperature showed the same activity as free Mn-SOD [44]. A recent study found that immobilization of recombinant human catalase on Au and Ag nanoparticles could decrease the activity of the enzyme [45]. In addition, immobilization of SOD on hollow silica nanospheres was found to result in a significant decrease of enzyme activity, to only 18.6% of the activity of native SOD [46]. When enzyme maltogenase was immobilized on AuNPs loss of activity was slight, while impact of AgNPs on activity of the enzyme was significant and low activity of the enzyme was determined [47].

The recombinant Mn-SOD on gold nanoparticles and in aqueous solution was stored at room temperature for 18 days. During this time, activity was measured repeatedly to monitor changes in enzyme activity. SOD was found to remain active throughout this period, and its activity decreased in a similar fashion for both the AuNP samples and the aqueous solution (Fig. 5). The same changes in activity were observed independently for nanoparticle size and percentage coverage. The presence of nanoparticles did not affect the activity of recombinant Mn-SOD throughout the experiment. Recent studies have shown that the immobilization of porcine pancreatic lipase on carboxyl functionalized silica-coated magnetic nanoparticles resulted in the prolongation of enzyme activity [48]. In addition, alcohol dehydrogenase immobilized on gold nanoparticles lost its activity more slowly than dehydrogenase in water solution when stored at 4 °C, and retained 50% of initial activity after 15 days [49]. Papain immobilized on porous magnetic nanoparticles after 28 days of storage in 4 °C retained nearly 85% of initial activity [50]. A similarly, Cu, Zn-superoxide dismutase loaded into liposomes demonstrated no significant loss of activity after 1 month storage at 4 °C [51]. These findings suggest that the type of nanoparticle influences the enzyme immobilized on it; however, it appears that immobilization of recombinant SOD on gold nanoparticles did not have any impact on enzyme activity.

**Fig. 6:**
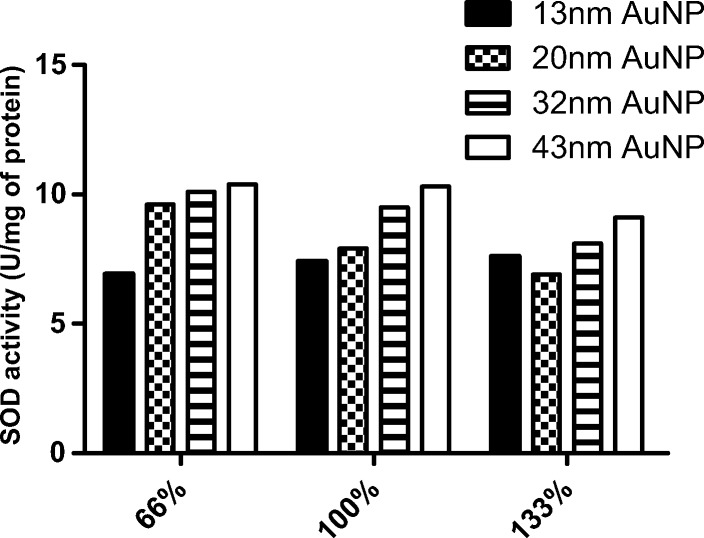
Activity of superoxide dismutase on gold nanoparticles at time point 0 according to the percentage coverage of nanoparticles

At a constant percentage of nanoparticle coverage, SOD activity was found to vary according to nanoparticle size (Fig. 6). In all coverage variants, the highest activity was demonstrated by the enzyme immobilized on the largest nanoparticles (43 nm) (Fig. 6). It could appear from the largest surface of nanoparticle and the highest amount of protein particles which are stabilized after immobilization. It has been demonstrated that the size of gold nanoparticles has slight influence on trypsin activity [52]. Recent studies have indicated that the size of gold nanoparticles influences on the activity of *Candida rugosa* lipase bound to them: smaller AuNPs appear to supplement the catalytic efficiency of lipase by enhancing its kinetic affinity (lower *K*_m_ values), but do not affect their relative activity [53].

**Fig. 7:**
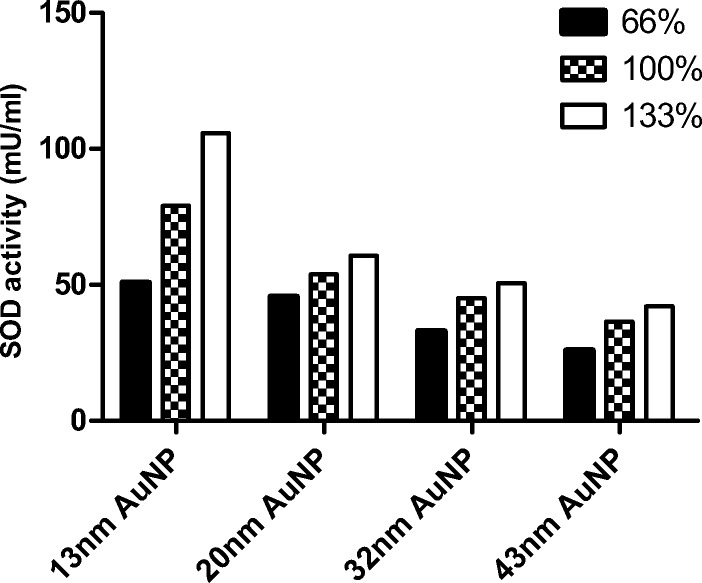
Activity of superoxide dismutase on gold nanoparticle in 0 time point according to the nanoparticle size

For each particle size and at the same concentration of nanoparticles, total SOD activity was found to be greatest for 133% nanoparticle coverage (Fig. 7), confirming that the enzyme particles on the nanoparticle surface are stabilized and active. Recent studies have demonstrated that the modulating activity of the protein-AuNP conjugate depended on the orientation of the protein and its density on the surface of the nanoparticle [54]. It has been also confirmed that specific activity of FMN-dependent enzyme nitroreductase (NfsB) immobilized on a self-assembled monolayer (SAM) of short poly ethylene glycol chains was dependent on surface concentration of the parallel oriented enzyme [55].

### Superoxide Dismutase Activity Test in the Presence of Silver Nanoparticles

**Fig. 8:**
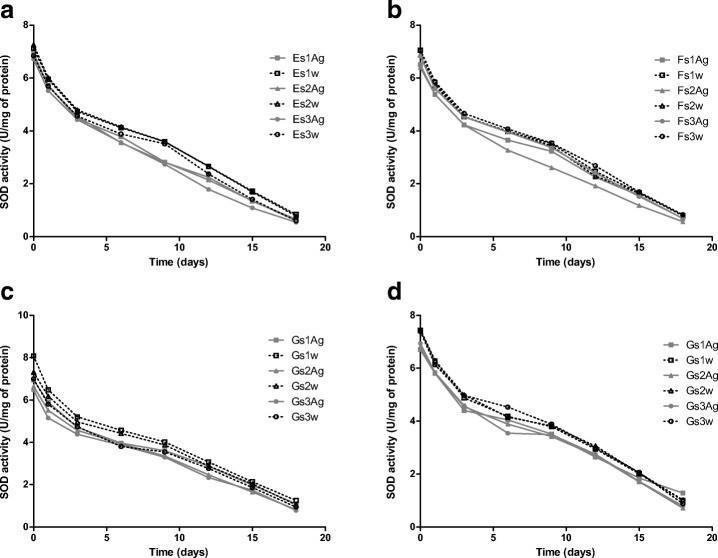
Activity of superoxide dismutase with silver nanoparticles (gray line) and in aqueous solution (black dotted line) in three variants of coverage (66% square, 100% triangle, 133% circle) for 18 days. **a** SOD with AgNPs of size 13 nm. **b** SOD with AgNPs of size 20 nm. **c** SOD with AgNPs of size 32 nm. **d** SOD with AgNPs of size 43 nm

Silver nanoparticles 13, 27, 33, and 45 nm in diameter were mixed with recombinant Mn-SOD to obtain 66, 100, and 133% of coverage of nanoparticles, as with the gold nanoparticles. Although SOD maintained its activity in the presence of silver nanoparticles, it was slightly lower than observed for aqueous solution for all nanoparticle size variants (Fig. 8). Recent studies have demonstrated that immobilization on silver nanoparticles could affect enzyme activity. Immobilization of alkaline phosphatase on 61-nm silver nanoparticles resulted in the loss of 33% of initial enzyme activity [56]. It was also determined that the functional activity of aA-Crystallin was significantly affected after adsorption onto the surface of AgNP [57]. Recent studies have shown that immobilization of lipase on cysteine modified AgNP permit to retain 66% of activity of free enzyme [58]. Immobilization of horseradish peroxidase on silver nanoparticles allowed 92% of enzyme activity to be maintained [59].

Superoxide dismutase with AgNPs was stored at room temperature for 18 days, during which time its activity decreased in a similar way to SOD in aqueous solution (Fig. 8). The presence of Ag nanoparticles in solution did not affect enzyme activity. It has been demonstrated that alcohol dehydrogenase immobilized on silver nanoparticles and stored at 4 °C for 15 days lost its activity to a similar degree as the enzyme stored in buffer solution [49].

**Fig. 9:**
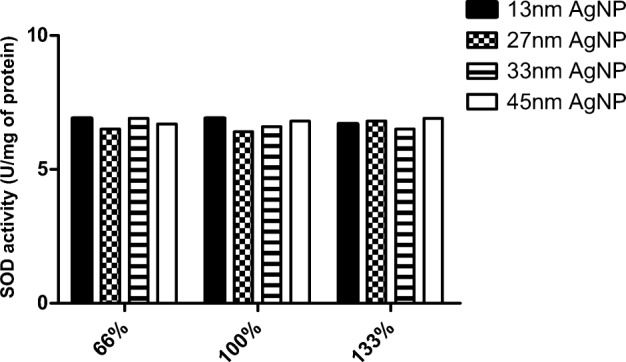
Activity of superoxide dismutase with silver nanoparticles at time point 0 according to the percentage coverage of nanoparticles

**Fig. 10:**
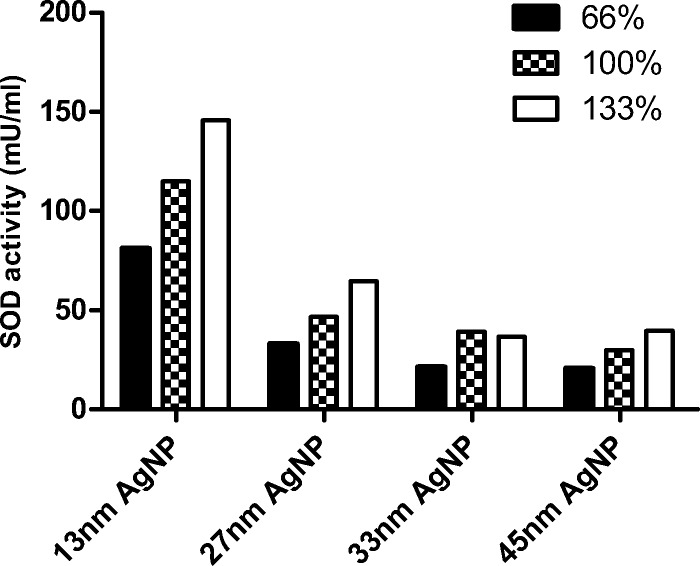
Activity of superoxide dismutase with silver nanoparticle at time point 0 according to the nanoparticle size

No difference in SOD activity was observed between different-sized particles with the same percentage of coverage (Fig. 9), unlike with gold nanoparticles (Fig. 6). Comparing total activity of SOD in colloids with silver nanoparticles shows the greatest SOD activity at highest nanoparticle coverage (133%), irrespective of nanoparticle size (Fig. 10).

Because SOD did not adsorb onto the surface of the nanoparticles, it is impossible to say whether these activity results are caused by immobilization. However, these findings do offer an insight into the interaction between the enzyme molecule and free silver nanoparticles.

## Conclusions

Recombinant human manganese superoxide dismutase (Mn-SOD) was produced in a bacterial expression system and was found to have activity after its isolation and purification. Gold and silver nanoparticles were successfully synthesized in four size variants of each type, without any aggregations or agglomerates. The recombinant SOD was immobilized on gold nanoparticles, as confirmed by SDS-PAGE, but not on silver nanoparticles; however, the presence of silver nanoparticles did not affect enzyme activity. Immobilization on gold nanoparticles had no influence on enzyme activity: its activity was the same as that demonstrated by SOD in aqueous solution for 18 days storage at room temperature. When bound to gold nanoparticles, SOD demonstrated the highest enzyme activity for colloids measuring 43 nm, and for particles with the greatest coverage. Those results suggest that size and the amount of protein immobilized on the metal nanoparticle have an impact on enzymatic activity in solution.

Our findings suggest that further studies are necessary for better understanding the interaction between metal nanoparticles and proteins. Nevertheless, the results appear to be interesting and useful for further research on the immobilization of enzymes on metal nanoparticles and its influence on enzymatic activity. Such conjugates of enzymes and gold or silver nanoparticles could be investigated as therapeutics in future in vivo studies.
